# Bioavailability of Glucosinolates and Their Breakdown Products: Impact of Processing

**DOI:** 10.3389/fnut.2016.00024

**Published:** 2016-08-16

**Authors:** Francisco J. Barba, Nooshin Nikmaram, Shahin Roohinejad, Anissa Khelfa, Zhenzhou Zhu, Mohamed Koubaa

**Affiliations:** ^1^Department of Food Science, Faculty of Science, University of Copenhagen, Copenhagen, Denmark; ^2^Nutrition and Food Science Area, Faculty of Pharmacy, Universitat de València, València, Spain; ^3^Department of Food Science and Technology, Faculty of Agricultural Engineering, Islamic Azad University of Sabzevar, Sabzevar, Iran; ^4^Burn and Wound Healing Research Center, Division of Food and Nutrition, Shiraz University of Medical Sciences, Shiraz, Iran; ^5^Sorbonne Universités, Université de Technologie de Compiègne, Laboratoire Transformations Intégrées de la Matière Renouvelable (UTC/ESCOM, EA 4297 TIMR), Centre de Recherche de Royallieu, Compiègne Cedex, France; ^6^School of Food Science and Engineering, Wuhan Polytechnic University, Wuhan, China

**Keywords:** glucosinolates, isothiocyanates, bioavailability, brassicaceae, myrosinase, processing

## Abstract

Glucosinolates are a large group of plant secondary metabolites with nutritional effects, and are mainly found in cruciferous plants. After ingestion, glucosinolates could be partially absorbed in their intact form through the gastrointestinal mucosa. However, the largest fraction is metabolized in the gut lumen. When cruciferous are consumed without processing, myrosinase enzyme present in these plants hydrolyzes the glucosinolates in the proximal part of the gastrointestinal tract to various metabolites, such as isothiocyanates, nitriles, oxazolidine-2-thiones, and indole-3-carbinols. When cruciferous are cooked before consumption, myrosinase is inactivated and glucosinolates transit to the colon where they are hydrolyzed by the intestinal microbiota. Numerous factors, such as storage time, temperature, and atmosphere packaging, along with inactivation processes of myrosinase are influencing the bioavailability of glucosinolates and their breakdown products. This review paper summarizes the assimilation, absorption, and elimination of these molecules, as well as the impact of processing on their bioavailability.

## Introduction

**Figure 1:**
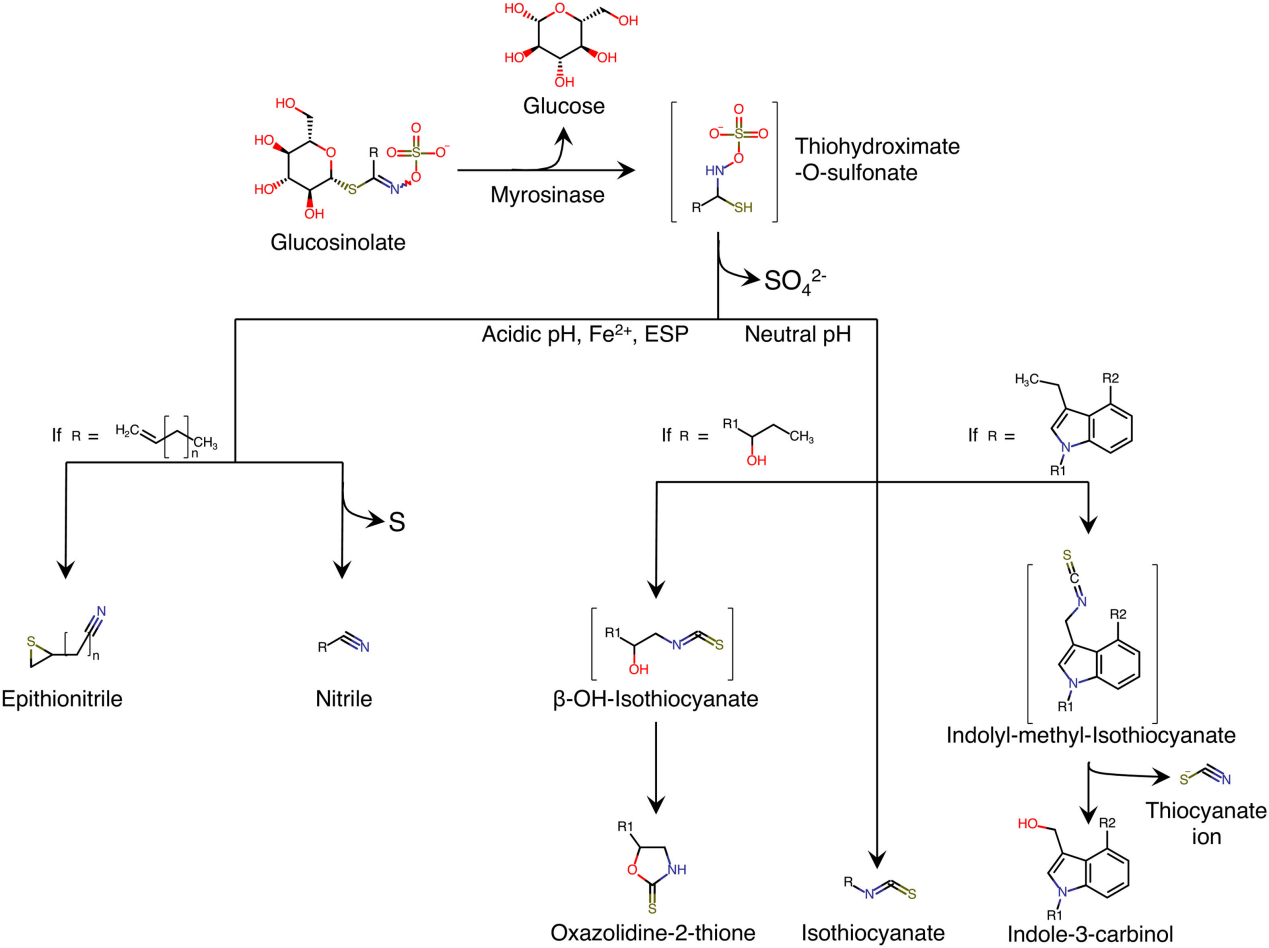
**Enzymatic hydrolysis reaction of glucosinolates and their breakdown products**. Adapted from Ref. (5). ESP, epithiospecifier protein; molecules between brackets indicate instable intermediate.

Glucosinolates are secondary metabolites synthesized by plants. They contain sulfur groups and are present in numerous species belonging to Brassicaceae family (1). Chemically, glucosinolates are composed of thiohydroximate-O-sulfonate group linked to glucose, and an alkyl, aralkyl, or indolyl side chain (R) (2) (Figure 1). More than 200 side-groups have been identified and cited in the literature (2–4).

Glucosinolates are relatively stable in plant cell. However, when the plant tissue containing glucosinolates is damaged, as is the case in the preparation (cutting, chopping, mixing) or chewing food, a β-thioglucosidase called myrosinase is released. The enzyme is normally stored separately from glucosinolates in different cells, or in different intracellular compartments, depending on the plant species (6). The hydrolysis of glucosinolate by myrosinase produces a molecule of β-d-glucose and an unstable aglycone; thiohydroximate-O-sulfonate (Figure 1). Spontaneous reorganization of this intermediate (chemical rearrangement of Lossen) results in the release of sulfate ion and in the formation of metabolites, the structures of which depend on the nature of the side chain (R) of glucosinolate, and the physico-chemical conditions of the medium (Figure 1). The formation of nitriles is favored by acidic pH and the presence in the environment of ferrous ions (Fe^2+^), and a particular plant protein called epithiospecifier protein (ESP) (7). In addition, the existence of terminal unsaturation in the side chain of the glucosinolate molecule leads to a distinct form of nitriles called epithionitriles. A neutral pH favors the formation of isothiocyanates (ITCs) (8). If the side chain has a β-hydroxyl function, ITCs cyclize spontaneously into oxazolidin-2-thione (1, 9, 10). Similarly, ITCs deriving from the hydrolysis of glucosinolates are unstable and they easily split into thiocyanate ion and indole-3-carbinol (Figure 1).

Isothiocyanates are highly reactive and present potent *in vivo* action as inducers of Phase II enzymes (11, 12). Numerous previous studies also reported their action as inhibitors of mitosis and stimulator of the apoptosis in human tumor cells (13–15). ITCs revealed also fungicidal, fungistatic, nematicidal, and bactericidal activities (16–18). Table 1 shows some biological activities of selected glucosinolate breakdown products. Due to the biological properties associated with glucosinolates and their breakdown products, especially ITCs, understanding, on the one hand, the absorption routes of these molecules and their metabolism and, on the other hand, the impact of processing parameters on their presence in food products is of great importance. This review is, thus, dealing to summarize both aspects.

**Table 1:** **Some biological activities of selected glucosinolate breakdown products**.

Biological activities	Glucosinolate breakdown products	Reference
Fungicidal effects	Allyl-ITC	(19–24)
	Alkenyl aliphatic ITCs (methyl-ITC, propenyl-ITC, butenyl-ITC, pentenyl-ITC) (propenyl-ITC, ethyl-ITC)	(22, 25)
	Benzyl-ITC	(21, 25–28)
	Butenyl-ITC	(19, 23)
	Glucoerucin-derived ITC	(29)
	Glucoiberin-derived ITC	(28)
	3-Indolylacetonitrile	(30)
	3-Methylsulfinylpropyl ITC	(28)
	Propenyl-ITC	(19, 31–34)
	Phenylethyl-ITC	(25, 35, 36)
	Sinalbin (p-Hydroxybenzylglucosinolate) derived-ITC	(37)
	Sinigrin (prop-2-enylglucosinolate)-derived ITC	(38)
	5-Vinyloxazolidine-2-thione	(39)
Bactericidal effects	Allyl-ITC	(40–45)
	Benzyl-ITC	(45)
	2-Phenylethyl-ITC	(45)
	4-hydroxybenzyl-ITC	(46, 47)
	Methyl-ITC	(42)
	4-(Methylsulfinyl)butyl ITC	(48–50)
	Phenyl-ITC	(51)
	Oxazolidinethiones	(52, 53)
Antiproliferative activities	Allyl-ITC	(54–59)
	Benzyl-ITC	(60–64)
	Indole-3-carbinol	(65)
	Indole ethyl-ITC	(66)
	4-Methylsulphinylbutyl-ITC	(48, 62, 67–77)
	7-Methylsulphinylheptyl-ITC	(67)
	Phenyl-ITC	(57)
	Phenylethyl-ITC	(70, 78–88)
	Phenylbenzyl-ITC	(89)
	Phenylmethyl-ITC	(89)

## Bioavailability of Glucosinolates and Their Breakdown Products

### Digestion in the Human Digestive Tract

Bioaccessibility represents the amount or fraction that is released in the digestive tract, from a food product, and is becoming available for absorption (90). The digestive transformations of the food material until assimilation and its enterocytic metabolism are also included in this definition. Bioavailability, on the other hand, is a subcategory of absorption and is the fraction of administered molecules that are absorbed and reach the circulation system (91). Numerous studies were focused on understanding the absorption and metabolism of glucosinolates and their breakdown products.

For instance, it has been demonstrated that indole-3-carbinol molecule, in acid medium, such as the gastric content, is condensed in the form of polycyclic aromatics (10, 92, 93). Generally, each glucosinolate can simultaneously provide different structures of aglycone. However, one of them is formed predominantly, depending on the structure of the side chain of glucosinolate and the environmental conditions. The breakdown products of glucosinolates are responsible for the typical aroma in cruciferous.

**Figure 2:**
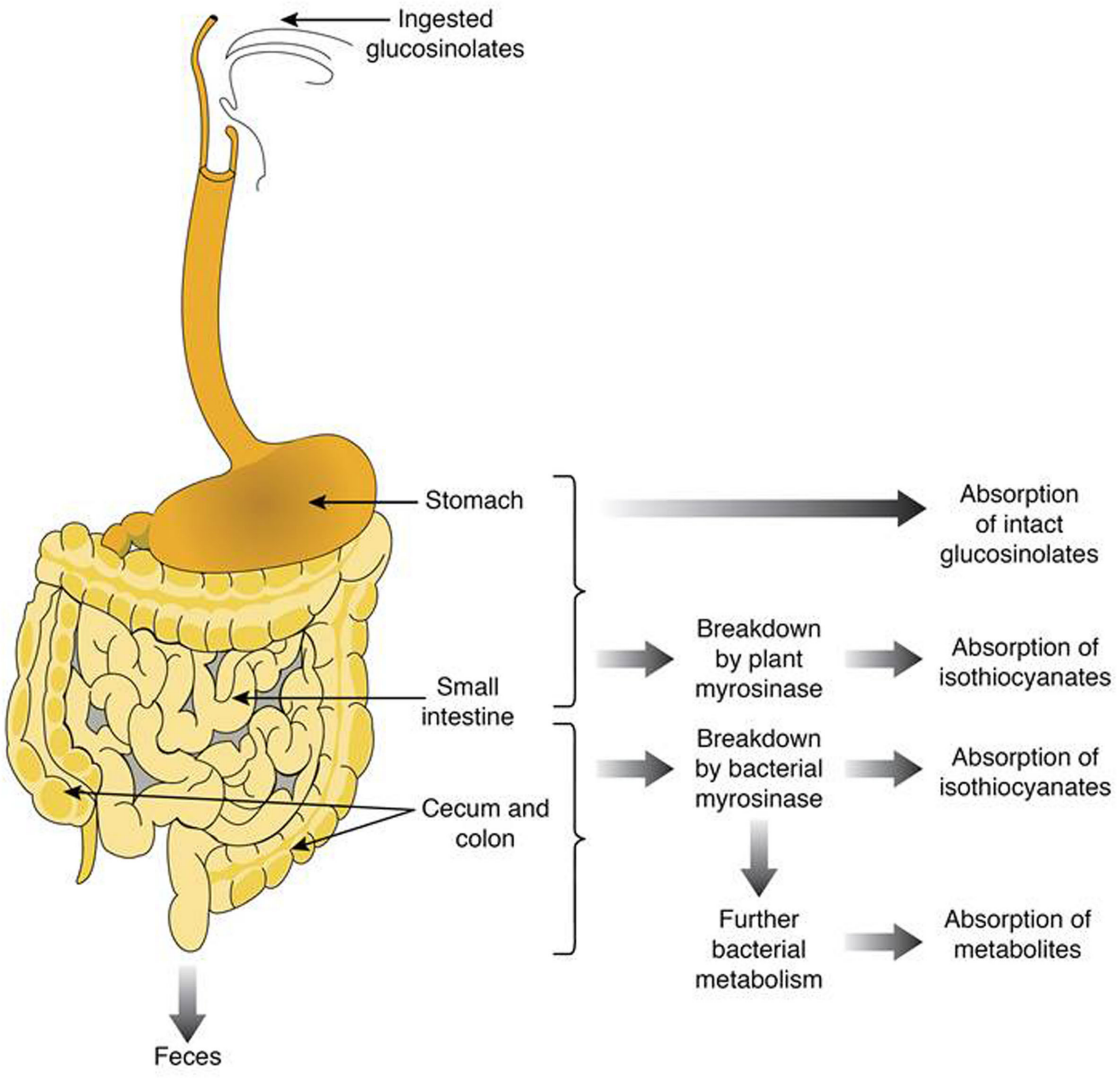
**Summary of the fate of glucosinolates and their breakdown products in the human gut**.

Cooking the plant material tends to denaturate the myrosinase. The intensity of the denaturation is particularly important when the applied temperature is high and the cooking time is long, whether by baking with water (94), steam (95, 96), or microwave (97). When myrosinase inactivated, glucosinolates transit to the colon, due to their hydrophilic nature (thioglucose and sulfate group), and are metabolized by the intestinal microbiota. Figure 2 shows the gastrointestinal parts where glucosinolates and their breakdown products are absorbed or metabolized. For instance, intact glucosinolates could be partially absorbed in the stomach, the remaining glucosinolates will transit through the gastrointestinal tract to reach the small intestine where they could be hydrolyzed by plant myrosinase, and the breakdown products could be absorbed. The remaining non-hydrolyzed glucosinolates will then transit to reach the colon where they could be hydrolyzed with bacterial myrosinase, and the generated breakdown molecules are absorbed or/and excreted.

It has been shown that the incubation of human feces in presence of pure glucosinolates or cruciferous vegetable juices, in which myrosinase was inactivated by heating, leads to the formation of ITCs (94, 98). These metabolites are also formed in the germ-free colon of rats, following the colonization with human intestinal bacteria, and feeding with a pure glucosinolate (99). In humans, urinary excretion of conjugated metabolites of ITCs was observed following the consumption of cruciferous cooked vegetables (94, 95, 97).

The formation of other breakdown products from glucosinolates by intestinal microbiota is very likely, but still poorly documented. The formation of amines from the secondary degradation of ITCs has been demonstrated after the incubation of human feces with glucosinolates (100). *Bifidobacterium* strains belonging to the human intestinal microbiota are able to *in vitro* metabolize the glucosinolates to nitriles (101) and traces of nitriles have been detected in the urine of rats fed with a pure glucosinolate (102). It is also known that many microorganisms are able to convert nitriles into ammonia and organic acids (103, 104). However, the formation of glucosinolate breakdown products other than ITCs in the human digestive tract requires more investigation.

### Absorption and Enterocytic Metabolism

Numerous works suggest that when active myrosinase is present in the food product, rapid hydrolysis of glucosinolates occurs in the proximal gut [small intestine (Figure 2)]. However, when myrosinase is inactivated (e.g., by cooking), intact glucosinolates could reach the distal gut (colon) where they are metabolized by bacterial enzymes. Nonetheless, the diversity of these enzymatic activities is associated with the generation of a wider range of metabolites than those so far identified.

In addition to myrosinase hydrolysis, some studies have shown that a small fraction could be absorbed in the native state by the lining of the small intestine. *In vivo*, this absorption results in the presence of native glucosinolates in urine up to 5% of the ingested dose (102). *Ex vivo* works, using intestinal loops isolated from rodents, suggest a passive or facilitated transport, independently of the glucose uptake mechanism (105).

### Post-Absorptive Metabolism, Tissue Distribution, and Elimination

Only the post-absorptive metabolism of ITCs has been extensively studied. The use of radiolabeled ITCs in rats indicates rapid absorption with a radioactive blood peak observed 3 h after ingestion (106, 107). Absorbed ITCs are conjugated to glutathione in the liver and excreted in the urine as mercapturic acid (*N*-acetyl-S-(*N*-alkylthiocarbamoyl)-l-cysteine), which account for 12–80% of the ingested dose of ITC (102, 106, 108, 109). In humans, the formation of mercapturic acids is the predominant metabolic pathway and the amount of mercapturic acid excreted is a good reflection of the amount of ITCs consumed.

Therefore, the mercapturic acids are used as biomarkers of intake and/or formed ITCs in the body (110, 111). Their urinary dosage in humans, following consumption of cruciferous vegetables, has shown that the bioavailability of ITCs depends on the respective contributions of plant myrosinase and intestinal microbiota in the hydrolysis of glucosinolates. Indeed, the excretion of mercapturic acids after consumption of raw cruciferous (action of plant myrosinase) accounts for 17–88% of the ingested dose of glucosinolates, depending on the molecule, the plant matrix (mustard, cabbage, watercress, broccoli, etc.), and the body (94, 95, 97, 110, 111).

When the vegetables are cooked (action of intestinal microbiota), this rate does not exceed 20% and the peak of mercapturic acids in urine occurs only after 12 h of ingestion, whereas it appears within 8 h when consuming raw cruciferous (94, 97, 107). All of these data suggest that the consumption of raw cruciferous leads to improved bioavailability of ITCs.

In parallel to the main pathway involving mercapturic acid, ITCs can follow minor excretion routes. In fact, overfed rats with radiolabeled ITCs excrete about 15% of radioactivity or in the form of CO_2_ in the expired air, or as unknown metabolites in feces. The presence of radioactivity in bile also indicates the existence of enterohepatic circulation of the metabolites. The tissues containing the highest concentration of radioactivity are the intestinal mucosa, the liver, the kidneys, and the bladder, followed by the lungs and the spleen. The brain and the heart contain very low concentration of radioactivity (106, 107, 109).

Concerning the other glucosinolate breakdown products, their assimilation by the body is still poorly understood. Similar to ITCs, nitriles and epithionitriles could be metabolized and excreted in the urine as mercapturic acids, whereas oxazolidine-2-thione and thiocyanate ion are directly excreted in urine (112).

The urine metabolites called dithiocarbamates, such as allyl-*N*-acetyl-l-cysteine (NAC), were measured in order to determine quantitatively the amount of ITCs metabolized after ingestion (113). Consumption of horseradish and broccoli led to 42–44% of glucosinolates metabolizing ITCs, and myrosinase inactivated in broccoli resulted in only 10–20% (113). The authors reported this behavior to the slow rate of glucosinolate breakdown with ongoing absorption. A large intestine *in vitro* model was employed to simulate digestion of pure sinigrin and myrosinase in freeze-dried Brussel sprouts. While allyl-ITC was detected in the run injected with sinigrin, no allyl-ITC was accumulated in those with Brussel sprouts. The optimal content of allyl-ITC was detected in 9–12 h, and only 10–30% of sinigrin was found to be converted into allyl-ITC, which might be due to the formation of other products of sinigrin hydrolysis, such as allylamine (98). To increase the bioavailability and bioaccessibility of glucosinolates, myrosinase activity of food matrix before its oral administration should be higher.

There is a growing interest in the health benefits of broccoli. Sulforaphane, the major bioactive component of broccoli, is an unstable ITC stored by the plant as glucoraphanin. Myrosinase enzyme release sulforaphane when the plant is crushed (114). Extraction of bioactive components during supplement formulation or heat processing of the plant material can destroy myrosinase.

## Impact of Processing on Glucosinolates and Their Breakdown Products

Processing of *Brassica* vegetables in domestic food preparation or during the industrial processing may influence significantly the contents of glucosinolates and consequently their health-protective capacities. Numerous factors, including post-harvest storage, preparation, and processing, have to be considered.

### Influence of Post-Harvest Treatments

The influence of post-harvest treatments on the quality and bioavailability of glucosinolates has been well documented in the literature (115–117). The main influencing parameters are the time, temperature, and the atmosphere packaging (118). In fact, the content of glucosinolates in cruciferous vegetables can be significantly affected by the storage conditions. This behavior has been demonstrated and investigated through numerous studies.

For instance, the effect of post-harvest and packaging treatments on glucoraphanin (4-methylsulfinylbutyl glucosinolate) concentration in broccoli (*Brassica oleracea* var. italica) has been investigated (119). Glucoraphanin is the glucosinolate precursor of anticancer ITC sulforaphane (4-methylsulfinylbutyl ITC). Results demonstrated that the concentration of glucoraphanin decreased by 55 and 56%, in broccoli stored during 3 days in open-air boxes and during 7 days in plastic bags, respectively. Moreover, this concentration was significantly higher in broccoli stored in controlled atmosphere packages than that stored under air treatment, and this during 25 days of storage. In addition, combining modified atmosphere packaging (MAP) with refrigeration at 4°C was most efficient than storage in air packages under the same conditions. In fact, no significant differences were observed in glucoraphanin concentration during 10 days of storage in MAP without holes at 4°C and in presence of two microholes at 20°C. The authors concluded that the best conditions of post-harvest storage of broccoli were under MAP and at 4°C.

In another work, the same species was subjected to analyze its content in indole glucosinolates, after harvesting (120). The plant material (inflorescences) was wrapped with low-density polyethylene (LDPE) film of 11-μm thickness, and stored during 7 days at 1°C. The overall idea was to simulate the conditions of a maximum transport and distribution period. Following this storage period, inflorescences were stored at 15°C during 3 days in order to simulate the retail sale period. Three predominant glucosinolates: glucoraphanin, glucobrassicin (3-indolylmethyl-glucosinolate), and neoglucobrassicin (1-methoxy-3-indolylmethyl-glucosinolate) were identified. At the end of cold and retail storage, the losses of glucosinolates were of 71 and 80%, respectively, compared to fresh harvest broccoli.

Recently, the content of glucosinolates in broccoli (*Brassica oleracea* L.) flower buds was determined during the post-harvest period as response to temperature and radiation treatments (121). Experiments were performed in order to simulate the commercial storage conditions from harvest until consumer consumption. For this purpose, the content of glucosinolates in broccoli flower buds was determined during a period corresponding to the refrigerated transport and retail. Two pre-storage periods (4 or 7 days) in the dark were tested at 0 or 4°C, followed by a storage period of 3 days at 10 or 18°C. The effect of radiation on glucosinolates content was performed by exposing the broccoli heads for 12 h/day, and this during the storage period, to three levels of either visible light (13, 19, or 25 μmol m^−2^ s^−1^) or a combination of visible light (19 μmol m^−2^ s^−1^) and UV-B irradiation (20 kJ m^−2^ d^−1^). For control experiments, samples were stored in the dark. Results indicated that the pre-storage at either 0°C or 4°C led to similar contents in glucosinolates, and this after 4 or 7 days. However, these pre-storage periods influenced significantly the content in glucosinolates during the storage period (at 10 or 18°C). The most affected molecules were total glucosinolates, total indolyl, total aliphatic glucosinolates, and all individual glucosinolate levels (with the exception of 4-methoxyglucobrassicin). It has been demonstrated that broccoli flower buds pre-stored during 7 days accumulated (after storage) more glucosinolates than that pre-stored during 4 days. The temperature of storage (10 or 18°C) was an influencing parameter on the glucosinolate contents.

The content of some molecules (i.e., 4-hydroxyglucobrassicin) was increased after storage at 18°C, whereas for some others, the content was increased after storage at 10°C (i.e., individual glucosinolates, total glucosinolates, and total aliphatic and indolyl glucosinolates). The effect of radiation showed that pre-storage at 0°C and during 7 days, followed by storage at 10°C during 3 days, and a radiation treatment with visible light of 25 μmol m^−2^ s^−1^, seems to influence significantly the content in aliphatic glucosinolates. Moreover, storage at 18°C combined with radiation treatment of visible light (25 μmol m^−2^ s^−1^) increased significantly the contents of indolyl 4-hydroxyglucobrassicin and 4-methoxyglucobrassicin in either flower buds or floret stalks.

In another study, the effect of storage in controlled atmosphere on the quality and health-promoting components of broccoli heads was investigated (122). Different controlled atmospheres (15% CO_2_ + 3% O_2_, 10% CO_2_ + 3% O_2_, 8% CO_2_ + 1% O_2_, 5% CO_2_ + 3% O_2_) were tested during a storage period of 100 days at 0°C. The contents of glucosinolates (sulforaphane and indole-3-carbinol) were determined before and after storage. Results demonstrated that the total contents of sulforaphane and indole-3-carbinol were higher in stored broccoli, compared to fresh ones. Moreover, 5% CO_2_ + 3% O_2_ was the most efficient atmosphere, compared to the other conditions, in terms of contents in glucosinolates. These works and others demonstrate the importance of storage conditions (time, temperature, and atmosphere packaging) on the contents of glucosinolates and, thus, their bioavailability.

### Influence of Preparation and Processing Conditions

Bioavailability of glucosinolates and their breakdown products could also be affected by culinary processing. For instance, the contents of glucosinolates in *Brassica* plants (broccoli, cauliflower, Brussel sprouts, and green cabbage) as well as the effect of storage and cooking conditions on stability of these compounds has been investigated (123). Results showed losses between 11 and 27% (detected partly as ITCs) in the content of glucosinolates after 7 days storage at ambient temperature and in a domestic refrigerator.

*Brassica* plants shredded finely demonstrated significant decrease in the contents of glucosinolates up to 75% over 6 h. On the other hand, thermal treatment by steam cooking, microwaving, and stir-frying did not induce significant changes in the contents of glucosinolates. However, boiling was more effective in reducing the levels of glucosinolates (approximately by 90%), by leaching into cooking water. The authors concluded that avoiding boiling of vegetables could increase the bioavailability of ITCs.

In another work, the contents of glucosinolates in chopped raw *Brassica* vegetables was investigated (124). Results demonstrated that aliphatic glucosinolates were partially breakdown in cabbage, whereas high level of indolyl glucosinolates was observed for chopped cabbage and broccoli stored at room temperature. After 48 h storage, chopped white cabbage showed higher contents (15 times more) in 4-methoxy- and 1-methoxy-3-indolylmethyl glucosinolates. Most of the glucosinolate contents (with the exception of 4-hydroxy- and 4-methoxy-3-indolylmethyl glucosinolates) were significantly reduced after chopping and storage. This reduction is mainly mediated by the action of myrosinase, which hydrolyzes the glucosinolates as reported above.

Therefore, many research groups have been interested in inactivating this enzyme in food products. In fact, the presence or not of active myrosinase directly impacts the degradation of glucosinolates. In order to consume intact glucosinolates, the inactivation of myrosinase is a key point during the food processing. The plant materials should be first harvested without damaging their tissues, which avoids the contact between glucosinolates and myrosinase, and then myrosinase is inactivated.

Several methodologies have been described in the literature to process this inactivation. In fact, it has been shown that the activity of myrosinase is sensitive to an increase in temperature beyond 80°C (125), and it is highly resistant to pressures up to 30 MPa (126). For instance, in a recent study, the composition of glucosinolates in *Brassica* was investigated during the post-harvest period, depending on the conditions and processing parameters (127).

Myrosinase activity was also evaluated on natural glucosinolates degradation. Experiments were conducted under different temperatures for 72 h, and processed using three cooking methods. Results showed that the temperature and the cooking method used, significantly impacted the contents of glucosinolates. Refrigerated samples contained the highest content in glucosinolates with 1.78 mmol/100 g dry weight. The authors concluded that steam method provided the less affected sample in term of glucosinolates content, compared to the conventional boiling water method, which led to the highest losses in the contents of glucosinolates (57 and 81% in *Brassica oleracea* and *Brassica rapa* cultivars, respectively).

There are two main processing methods for inactivating myrosinase: thermal (i.e., heating, cooking, blanching with steam, heating by microwaves or radio frequency) and non-thermal (i.e., high hydrostatic pressure, pulsed electric fields). For instance, in the conventional scheme of oilseed crushing, the enzyme is released during flaking of the seeds. A rapid heating of the flakes up to 80–90°C before pressing allows the inactivation of myrosinase. However, the operating conditions and the time interval between the release of the enzyme and its inactivation are sufficient to initiate the hydrolysis of glucosinolates.

It has been demonstrated that this type of heat treatment is efficient only for a partial inactivation of myrosinase when the water content is less than 8% (128). It was also observed that during the extraction of glucosinolates from rapeseed by water at 80°C, the activity of myrosinase was still significant, and it was necessary to operate at more than 90°C to avoid the degradation of glucosinolates. The use of a mixture of water/methanol or water/ethanol was more efficient to inactivate myrosinase at a temperature around 70°C (129).

The use of microwaves allowed shortening the processing time and decreasing the heating temperature to inactivate myrosinase (130). Autoclaving, on the other hand, combines the effect of temperature and pressure for more efficiency. High pressure (70 MPa) without treatment was shown to be efficient to inactivate myrosinase (131). Autoclaving with ethanol could be an alternative to steam inactivation, which avoids the loss of glucosinolates with water. Recently, other technologies, such as supercritical CO_2_ and ultrasounds, have been successfully tested as non-thermal inactivation methods of enzymes and could, thus, be suitable for the inactivation of myrosinase (132, 133). The use of these non-conventional technologies and others has been well reviewed recently (4).

Although the common inactivation method of myrosinase is by heating, selecting the most appropriate process is crucial to maintain high content of glucosinolates in the food product. As shown in Table 2, steaming is the best applied technique of heat treatment compared to conventional boiling or microwaving as it preserves high content of glucosinolates (depending on prior culinary preparations, such as cutting), and at the same time inactivates myrosinase. However, a compromise between increasing the bioavailability of glucosinolates (with myrosinase inactivation; for example, by steaming), and enhancing the flavors of the commercialized foods by generating breakdown products (without myrosinase inactivation) should be taken into account.

**Table 2:** **Impact of cooking conditions on glucosinolates and their breakdown products**.

Cooking conditions	Main findings	Reference
Boiling or steaming for 10 min	Reducing sinigrin by 9.6 and 29.1% in steamed and boiled cauliflower	(134)
Blanching, microwaving, or steaming cabbage for up to 10 min	Blanching decreased glucosinolate and S-methylmethionine levels, whereas microwaving or steaming preserved them	(135)
Steaming for 10 min, boiling for 15 min, and high-pressure cooking for 7 min	Losses between 20–33% and 45–60% in pressure treatment and boiled vegetables, respectively. Breakdown products of aliphatic glucosinolates decreased from 5 to 12% in steamed, 18 to 23% in pressure-cooked, and 37 to 45% in boiled samples	(136)
Boiling Brussels sprouts at 100°C for 5, 15, and 30 min	The presence of seven breakdown products (indole-3-acetonitrile, indole-3-carbinol, ascorbigen, 3,3′-diindolylmethane, 3-butenylnitrile, 4-methylsulfinylbutanenitrile, and 2-phenylacetonitrile) after boiling	(137)
Boiling for 5 min. Stir-frying at 130°C for 5 min. Microwaving (450 W) for 5 min. Steaming for 5 min	Compared with fresh-cut red cabbage, all cooking methods were found to cause significant reduction in total glucosinolates contents	(138)
Boiling in water with a cold start (25°C); boiling with a hot start (100°C); and steaming	Steaming showed an increase in the amount of total glucosinolates (+17%). Boiling-hot start (−41%) and boiling-cold start (−50%) reduced total glucosinolates	(139)
Cutting (2-inch pieces) and then hot water blanching at 66, 76, 86, and 96°C for 145 s	Blanching at ≥86°C inactivated peroxidase, lipoxygenase, and myrosinase. Blanching at 76°C inactivated 92% of lipoxygenase activity, and leads to 18% loss in myrosinase-dependent sulforaphane formation	(140)
Radio frequency cooking in oven transferring 180 kJ. Steaming for 8 min at 100°C	Increasing glucosinolates from 10.4 μmol g^−1^ DW in fresh broccoli to 13.1 and 23.7 μmol g^−1^ DW after radio frequency cooking and steaming, respectively	(141)
Cooking at 100°C for 8 and 12 min	Limited thermal degradation of glucoraphanin (less than 12%) was observed when broccoli was placed in vacuum-sealed bag	(142)
Cutting broccoli (15 cm long). Boiling for 3.5 min at 100°C. Low pressure (0.02 MPa) steaming at 100°C, 5 min. High-pressure (0.1 MPa) steaming for 2 min. Under vacuum treatment at 90°C for 15 min. Microwaving at 900 W, 2.5 min. Vacuum-microwaving (−98.2 kPa) at 900 W, 2.5 min	Boiling and under vacuum processing induced the highest glucosinolate loss (80%), while low-pressure steaming, microwaving, and vacuum-microwaving showed the lowest (40%) loss	(143)
Blanching broccoli for 30, 90, and 120 s. Stir-frying at 100–130°C for 90 s. Microwaving at 800 W for 90 s	Blanching at 120 s decreased total glucosinolates by 36%, stir-frying and microwaving decreased them by 13–26%	(144)
Boiling and steaming Portuguese cabbage for 12 min, and for 15 min for the other *Brassica*. Microwaving at 850 W, 8 min	Steaming contributed to the higher glucosinolates preservation, whereas boiling water led to higher losses (57% in *Brassica oleracea* and 81% in *Brassica rapa* cultivars)	(127)
Microwaving at 1100 W, steaming and boiling. Cooking times were 2 or 5 min	Steaming resulted in higher retention of glucosinolates, while boiling and microwaving resulted in significant losses	(145)
Boiling, high-pressure cooking, and steaming for up to 15 min	Better preservation of glucosinolates with steaming. Similar losses (64%) after boiling and high-pressure cooking	(146)
Microwaving (590 W, 5 min), frying (180°C, 5 min), frying (3 min)/microwaving (2 min), steaming (5 min), and baking (200°C, 5 min)	Significant modifications of total aliphatic and indole glucosinolates by all cooking treatments, except for steaming	(147)

## Priority Research Needs

One of the most important points to study, related to the assimilation and metabolism of glucosinolates and their breakdown products, is the *in vivo* studies about the mechanisms of interaction between these molecules and their target tissues. For this purpose, experiments must be performed with human volunteers, in which defined *Brassica* vegetables will be preferably used. For instance, dose–response behavior should be investigated for both the benefits and the side effects associated with the consumption of glucosinolates and their breakdown products. This relationship could be properly quantified by using biomarkers (other than mercapturic acid), which is a challenging achievement. Moreover, increasing the studies in breeding *Brassica* vegetables is of great importance as it leads to improving the availability of glucosinolates by the generation of high accumulating plants in these molecules.

Another important point to take into account is the investigation of the interaction between glucosinolates and their breakdown products, on the one hand, and the other food constituents of the whole diet, on the other hand. Studies were mostly focused on the assimilation and metabolism of individual molecules, whereas many factors may influence the digestive and post-absorptive metabolism of glucosinolates and their derivatives and, therefore, the excretion routes and tissue disposition. For instance, it has been shown that dietary fibers may modulate the assimilation of glucosinolates (148), and this is very important as most of the time, consumed foods have complex and different compositions, which influences the digestive fate of these compounds. Finally, the degradation rate of glucosinolates during the processing of food products is insufficiently understood, as the breakdown products are generated simultaneously, and the degradation depends on the process applied. In this line, establishing predictive models to study such phenomena is of great importance. These models should be associated with those predicting the bioavailability and biological activities in humans.

## Conclusion

Bioavailability of glucosinolates and their breakdown products depends on many factors, including the inactivation or not of myrosinase, the processing and storage conditions, and the association with other food constituents. It is well known that consumption of *Brassica* vegetables is associated with anticarcinogenic effects and other beneficial biological activities of the breakdown products. Numerous studies have described the assimilation and metabolism of both glucosinolates and their derivatives, however, the literature is lacking the description of detailed mechanisms and models associated with these phenomena. With regard to the beneficial effects of glucosinolates, in addition to breeding, transgenic plants could also be a possible way to enhance specific molecules by either overexpressing or inactivating genes, or even more, cloning regulatory factors, which is insufficiently studied up to the present.

## Author Contributions

FB, NN, SR, AK, ZZ, and MK have been involved in checking literature, writing the paper, and reviewing the final version.

## Conflict of Interest Statement

The authors declare that the research was conducted in the absence of any commercial or financial relationships that could be construed as a potential conflict of interest.

## Abbreviations

ESP, epithiospecifier protein; ITCs, isothiocyanates; MAP: modified atmosphere packaging; NAC, *N*-acetyl-l-cysteine.
